# Draft Genome Sequence of Aspergillus ochraceus Strain DY1, a Lignin-Degrading Fungus Isolated from Wood Rot

**DOI:** 10.1128/mra.01278-22

**Published:** 2023-03-06

**Authors:** Menaka Devi Salam, Namdol Nilza, Ajit Varma

**Affiliations:** a Amity Institute of Microbial Technology, Amity University Uttar Pradesh, Noida, Uttar Pradesh, India; University of Maryland School of Medicine

## Abstract

Aspergillus spp. are known for their lignin-degrading ability and also for the degradation of complex aromatic compounds. In this paper, we present the genome sequence of Aspergillus ochraceus strain DY1, which was isolated from rotten wood in a biodiversity park. The total genome size is 35,149,223 bp, including 13,910 hits of protein-encoding genes, with a GC content of 49.92%.

## ANNOUNCEMENT

Aspergillus ochraceus strain DY1 was isolated from rotten wood of Acacia nilotica (gum Arabic tree) that had been collected from a biodiversity park in Noida, Uttar Pradesh, India (28.5109N, 77.4005E). The isolation procedure involved initial surface sterilization of wood chunks with 95% ethanol, followed by suspension of 10 g of wood pieces in 100 mL of sterile normal saline (0.85%). An inoculum of 100 μl of the suspended mixture was spread on potato dextrose agar (PDA) and incubated at 28°C for 7 days. On the basis of screening results for the isolates, i.e., formation of a clear zone around the isolate on PDA containing aniline blue dye (0.05 g/L) and decolorization of Kraft lignin (KL) in minimal medium within 3 days of incubation, fungal isolate DY1 was selected as a potential lignin degrader. Further, through internal transcribed spacer (ITS)-5.8S rRNA gene sequencing analysis, it was found that the isolate was Aspergillus ochraceus. Whole-genome sequencing was carried out in order to understand the enzymatic functions and the pathway underlying the efficient lignin-degrading activity.

Aspergillus ochraceus strain DY1 was grown on minimal medium supplemented with 0.02% (wt/vol) KL as the sole carbon source for 7 days at 28°C. Genomic DNA (gDNA) was isolated using the AllPrep fungal DNA isolation kit (Qiagen), and the purity and concentration were analyzed using a NanoDrop 2000 spectrophotometer. The gDNA was randomly sheared into short fragments, which were end repaired, A-tailed, and further ligated with Illumina adapters. The fragments with adapters were PCR amplified, size selected, and purified. A paired-end gDNA library was prepared using an Illumina DNA preparation kit and was sequenced using an Illumina NovaSeq 6000 instrument. The whole-genome sequencing resulted in 4.2 GB of raw data, which was processed using FASTX-Toolkit v0.0.13 (Cold Spring Harbor Laboratory, Cold Spring Harbor, NY, USA) (1). All 150-bp paired-end raw reads from the Illumina sequencer were quality checked for low-quality bases, and adapter sequences were removed. A read was retained if at least 70% of the bases had scores of ≥Q30. The minimum read length accepted was 50 nucleotides. The processed data totaled 3.8 GB. All processed reads were assembled into contigs without any reference (*de novo*) using Velvet v1.2.10 software (2). Assembly was tried with various hash lengths (*k*-mers), and 69 was selected as the best hash length. The best *k*-mer is decided on the basis of various parameters, such as number of contigs, total number of reads used, total contig length, and number of non-ATGC characters. For scaffolding and gap closing, SSPACE-standard v3.0 (3) and GapCloser-bin v1-12-r6 (4), respectively, were used. The raw reads totaled 28,304,202 bp, with each read being 150 nucleotides. The expected sequencing depth was 120×, and the percentage of reads used was 97.13%. The draft genome sequence is 35,149,223 bp long, assembled into 211 contigs, with an *N*_50_ value of 1,168,992 bp. The genome size is similar to the reported sizes of other Aspergillus ochraceus genome assemblies (5, 6). Gene prediction and annotation were performed using the AUGUSTUS tool (7). A total of 13,910 hits of protein-encoding genes were obtained and were subjected to a homology search against proteins of Aspergillus spp. in the UniProt database (8). Results were parsed for best hits and were annotated with the Gene Ontology information, which revealed 470 genes for the metabolism of carbohydrates and aromatic compounds that could be linked to lignocellulose degradation.

### Data availability.

The whole-genome sequence of Aspergillus ochraceus strain DY1 has been deposited in DDBJ/ENA/GenBank under accession number JAPDNX000000000, BioProject accession number PRJNA873887, and BioSample accession number SAMN30503997, with SRA accession number SRR22188064.
